# Persistence with treatment for Wilson disease: a retrospective study

**DOI:** 10.1186/s12883-019-1502-4

**Published:** 2019-11-12

**Authors:** Wojciech Masełbas, Anna Członkowska, Tomasz Litwin, Maciej Niewada

**Affiliations:** 1 Department of Experimental and Clinical Pharmacology, Medical University of Warsaw, Warsaw, Poland; 2 2nd Department of Neurology, Institute of Psychiatry and Neurology, Warsaw, Poland

**Keywords:** Wilson disease, Persistence, Treatment outcome

## Abstract

**Background:**

Wilson disease (WD) is genetically induced failure of copper metabolism which can be successfully treated with pharmacological agents. The prognosis for survival in most WD patients is favorable if diagnosis and anti-copper treatment are provided early. Many observations imply that persistence with drug treatment is generally low in patients with chronic diseases, which impact the treatment effectiveness, but such results are very limited in WD. The aim of our study was to assess persistence with treatment among WD patients, to analyze its effect on patient outcome and to identify factors that might be related to persistence.

**Methods:**

170 newly diagnosed, symptomatic patients with WD who started treatment between 1995 and 2005 were analyzed retrospectively to assess treatment non-persistence, which was defined as at least one reported break of more than 3 months or minimum two breaks lasting longer than 2 months. Results were further analyzed according to selected clinical variables.

**Results:**

Only 74.1% of patients were persistent with treatment during the mean 11.7 years of follow up. Treatment persistence closely impacted positive clinical outcomes. In patients classified as persistent, improvement and lack of WD progression were observed more often compared to those classified as non-persistent (29.4 and 68.3% vs. 2.3 and 45.5%; *p* < 0.001, respectively). In contrast, non-persistent patients presented more often with worsening WD than persistent patients (52.3% vs. 2.4%). Type of WD treatment, gender, phenotypic presentation, adverse events and duration of treatment were not related to treatment persistence. Higher or upper/post-secondary education and a supportive family attitude towards treatment were the most important factors related to persistence.

**Conclusions:**

One quarter of WD patients were not taking anti-copper treatment regularly and this had an important negative effect on clinical outcome. Family support played an important role in treatment persistence.

## Background

Wilson disease (WD) is genetically induced failure of copper metabolism caused by mutations in the *ATP7B* gene located on chromosome 13 and is inherited in an autosomal, recessive pattern [1, 2]. Mutated *ATP7B* leads to dysfunction of the copper-transporting adenosine triphosphatase that it encodes, which results in reduced incorporation of copper into ceruloplasmin and lower biliary excretion of this microelement. Copper ingested in food initially accumulates in the liver and after exhausting its storage capacity infiltrates the brain, cornea (Kayser-Fleischer ring), and kidneys [1–3]. If untreated, WD usually leads to death within several years from the development of symptoms [1–5]. Onset of symptoms is mainly at the pediatric and adolescent age, but it is not infrequent to find patients first have symptoms during adulthood [1–4]. The prevalence of WD in European populations varies between 12 and 29 cases per 100,000 [1–4].

WD is one of the few inborn metabolic diseases that can be successfully treated [1, 2]. Therapy is based on products that promote urinary excretion of copper (D-penicillamine and trientine) or prevent its absorption via the gastrointestinal tract (zinc salts) [1–3]. However, there is a lack of high-quality evidence to estimate the relative effects of available treatments [1–3, 5–7]. Patients with hepatic presentation of WD are more often treated by D-penicillamine while zinc seems to be preferred for the treatment of pre-symptomatic and neurological patients [1, 2, 8]. While there appears to be no obvious differences in the clinical efficacy of available treatments, the choice of drug is most often based on tolerability profiles. In the majority of cases, early diagnosis and treatment help to prevent irreversible copper damage but treatment should continue over the whole lifespan after the diagnosis. Unfortunately, in some patients, the disease is relentlessly progressive even despite of treatment [1, 2, 8].

There are many observations implying that concordance with drug treatment including compliance (using the prescribed dose), persistence (continuation in drug use), and adherence (taking medication as prescribed in relation to time of intake and meals) in patients with chronic diseases is especially low and that this has a strong negative impact on treatment outcome [8–16]. However, apart from clinical studies prospectively analyzing medicine dose dispensations and the completion of patient diaries, assessment of compliance and adherence are very difficult to perform. In contrast, persistence can be assessed based on patient questionnaires and medical history according to treatment breaks taken by the patient [16].

The aim of this observational study was to assess treatment persistence among patients with WD as well as to attempt to identify factors that may be related to persistence.

## Methods

### Patients and assessments

The study was approved by the Bioethics Committee of the Institute of Psychiatry and Neurology in Warsaw, Poland. The conduct of this project was compliant with principles outlined in the Declaration of Helsinki.

The initial target population included 219 clinically symptomatic WD patients registered in the database of Institute of Psychiatry and Neurology at Warsaw that were diagnosed with WD and started the treatment between January 1995 and December 2005. Pre-symptomatic patients were the subject of an earlier analysis included into a different paper from our team [17]. WD diagnosis was established by the following criteria: medical history, physical examination, abnormal serum ceruloplasmin and copper levels results, increased 24-h urinary copper excretion, genetic analysis for known mutations and with ^64^Cu radioactive test (in doubtful cases) [1, 2, 7, 17]. Patients were defined as being symptomatic if they had clinical signs and symptoms of WD at diagnosis. Just after diagnosis, the patient received anti-copper treatment with D-penicillamine or zinc sulphate, the two main treatments used in our center.

At the time of study conduct from the initial group of 219 patients, 16 were reported dead, 10 had received a liver transplant, 1 was considered as lost to follow up and 2 refused to participate in the study. The remaining 190 were asked between February and June 2012 to complete a detailed questionnaire collecting information on: persistency of drug use, cause of non-persistence, adverse events (AE), achieved education level, family knowledge about WD, and family support provided to study participants.

The questionnaire included questions based on already validated scales: *Medication Adherence Questionnaire (MAQ), Brief Adherence Rating Scale (BARS), Medication Adherence Rating Scale (MARS)* and items to assess persistence with drug treatment, with persistence defined as continuing drug use [18–21]. Patients were classified as persistent or non-persistent by the attending physicians (AC, TL, WM) after detailed interview, and analysis of patient questionnaires, refills, and drug consumption. Patients were considered non-persistent if they had at least one reported break of more than 3 months or a minimum of two breaks lasting longer than 2 months within the period of treatment.

In addition to patient questionnaires, we also collected available medical records related to demographic and clinical characteristics, with a special focus on prescribed medication, changes in treatment, reported AEs and clinical outcome. The majority of subjects from the target population frequently visited our Institute for consultation even if their regular medical care was provided in other medical facilities. From the reminder, data were obtained by telephone contacts. Responses of study subjects were verified with information from medical history of each study participant.

According to clinical symptoms and signs, patients were classified as having the hepatic and neurological form of WD using the predominance symptoms scoring system based on the presence and intensity of individual signs of WD at diagnosis and as used previously [7, 17]. The hepatic assessment included a structured interview with questions about fatigue, weight loss, leg edema, jaundice, abdominal swelling, hematemesis, hemorrhages, and fulminant liver failure. Routine laboratory examinations included measurements of aminotransferases, bilirubin, international normalized ratio, serum albumin, and abdominal ultrasonography. The upper normal limit for alanine and aspartate transaminases was 40 IU/L.

To assess neurological state, a scale ranging from 0 to 3 (0, no symptoms; 1, light; 2, mild; 3, severe neurological signs) was used. Tremor of head, limbs, other involuntary movements, rigidity, speech, writing, and gait were evaluated [7, 17]. Patients classification was performed and verified by the same neurologists (AC, TL).

We analyzed the impact of treatment persistence on the clinical outcome of treated patients. Clinical outcomes of treatment were assessed by the attending physicians using standardized criteria. For hepatic symptoms, improvement was defined as liver enzymes reaching normal values or decreasing to a value lower than double the upper limit of normal value. Worsening was defined as significant increase of hepatic signs and/or symptoms, or liver enzyme levels exceeding double baseline values. For neuropsychiatric symptoms, the gain or loss of one point, based on our neurological scale, was regarded as improvement or deterioration [7, 17].

Based on studied literature, we also assessed persistence predictors with treatment like the type of WD treatment (D-penicillamine vs. zinc sulphate), gender, phenotypic presentation of WD, AEs, the length of WD treatment as well as patients achieved education (vocational vs. upper secondary/post-secondary vs. higher education), and the knowledge and support of family members about WD.

### Statistical analysis

Obtained data were reported with descriptive statistics and analyzed by two-tailed Fisher’s exact test, chi-square test, Mann-Whitney test and unpaired Student’s t-test were appropriate. All data were analyzed in relation to treatment persistence (yes/no).

## Results

Of the 190 patients asked to complete the detailed questionnaire, responses were obtained from 172 patients (accrual rate 90.5%); however, due to incomplete data in two surveys, the final analysis was conducted on 170 subjects. The demographic and clinical characteristics of these patients are presented in Table 1. The study population were 54.7% female, with a mean age of 37.5 years and a mean age at diagnosis of 25.0 years.

**Table 1:** Demographic and clinical characteristics

Variable, *n* = 170	Values
Male:female ratio, *n* (%)	77 (45.3):93 (54.7)
Age, mean ± SD (years)	37.5 ± 10.4
Age at diagnosis, mean ± SD (years)	25.0 ± 10.0
Time from symptom onset, mean ± SD (months)	15.9 ± 14.2
Duration of treatment, mean ± SD (years)	11.7 ± 3.2
Predominant clinical form at diagnosis, *n* (%)	
Hepatic	79 (46.5)
Neurological	91 (53.5)
Type of initial treatment, *n* (%)	
D-penicillamine	98 (57.6)
Zinc sulphate	72 (42.4)
Type of treatment during assessment, *n* (%)	
D-penicillamine	76 (44.7)
Zinc sulphate	94 (55.3)
Persistence in drug use, *n* (%)	
Yes	126 (74.1)
No	44 (25.9)
Adverse events, number of subjects (%)	53 (31.2)
Outcome of treatment (at the time of assessment), *n* (%)	
Improvement	38 (22.4)
No change	106 (62.4)
Worsening	26 (15.3)

*SD* standard deviation

The mean (SD) length of treatment was 11.7 ± 3.2 years, with most patients (57.6%) starting treatment with D-penicillamine, and the rest with zinc sulphate. Twenty-seven out of 76 patients (35.5%) who were taking D-penicillamine at the time of the assessment reported AEs related to drug treatment. Most frequently occurring AEs were rash (11 cases), gastrointestinal problems (3), abdominal pain (2), leucopenia (2), and proteinuria (2). Out of 94 patients taking zinc sulphate, AEs occurred in 26 patients (27.7%). Most often reported AEs were nausea and vomiting (15 cases) and abdominal pain (11 cases).

The persistence rate with WD treatment, among analyzed patients, was 74.1%. Treatment persistence had a significantly positive impact on clinical outcome (Table 2). In patients classified as persistent, positive outcomes were observed more frequently: improvement (29.4%) and lack of progression of disease (68.3%) vs. those who made breaks in drug intake (2.3%; and 45.5%, respectively). Out of 126 patients using prescribed medication persistently, only 3 deteriorated (2.4%) in comparison to 23 out of 44 not taking the medication persistently (52.3%). This outcome was observed regardless of predominant symptoms of disease (data not shown). We did not find any difference in treatment effectiveness between D-penicillamine and zinc sulphate in the patients analyzed (*p* = 0.85; data not shown). Subsequently, we found no substantial differences between treatment persistence rate among patients that were taking D-penicillamine and zinc sulphate (75.0% vs. 73.4%; *p* = 0.81). There was no impact on treatment persistence when analyzed by gender (68.8% persistence rate in males vs. 78.4% in females; *p* = 0.15), phenotypic presentation (72.1% persistence rate in the hepatic group vs. 75.8% in the neurological group; *p* = 0.59), AE occurrence (*p* = 0.42), or duration of WD treatment (*p* = 0.81) (Table 3).

**Table 2:** Treatment outcome by treatment persistence

	Treatment outcome, *n* (%)		
	Improvement (*n* = 38)	No change (*n* = 106)	Worsening (*n* = 26)
Persistent drug use (*n* = 126)	37 (29.4)	86 (68.3)	3 (2.4)
Non-persistent drug use (*n* = 44)	1 (2.3)	20 (45.5)	23 (52.3)
*P* value for comparison of persistent vs. non-persistent drug use		*p* < 0.001

**Table 3:** Persistence breakdown by symptoms, adverse events, drug use, knowledge of family members about Wilson disease (WD) and position towards treatment

Description	Persistent	Non-persistent	*P* value for persistent vs. non-persistent
Symptoms, *n* (%)			
Hepatic (*n* = 79)	57 (45.2)	22 (50.0)	0.59
Neurological (*n* = 91)	69 (54.8)	22 (50.0)	
Gender, *n* (%)			
Male (*n* = 77)	53 (42.1)	24 (54.5)	0.15
Female (*n* = 93)	73 (57.9)	20 (45.5)	
Adverse events, *n* (%)			
Yes (*n* = 61)	43 (34.1)	18 (40.9)	0.42
No (*n* = 109)	83 (65.9)	26 (59.1)	
Drug used, *n* (%)			
D-penicillamine (*n* = 76)	57 (45.2)	19 (43.2)	0.81
Zinc sulphate (*n* = 94)	69 (54.8)	25 (56.8)	
Treatment duration, mean ± SD, years	11.7 ± 3.2	11.6 ± 3.0	0.81
Educational level, *n* (%)			
Vocational education	29 (23.0)	11 (25.0)	
Upper secondary/post secondary	51 (40.5)	27 (61.4)	0.01
Higher education	46 (36.5)	6 (13.6)	
Family knowledge about WD, *n* (%)			
Minimal	4 (3.2)	4 (9.1)	
Little	21 (16.7)	12 (27.3)	0.14
Moderate	42 (33.3)	12 (27.3)	
Good	59 (46.8)	16 (36.4)	
Family position towards treatment, *n* (%)			
Negative	0	0	< 0.001
Neutral	16 (12.7)	18 (40.9)	
Supportive	110 (87.3)	26 (59.1)	

*SD* standard deviation

Overall, 52 (30.5%) patients received higher education while 78 (45.8%) received upper secondary/post-secondary education and 40 (23.5%) patients received vocational education. Education appeared to affect persistence rate, such that persistent patients were more likely to report higher education with no difference for vocational education level (Table 3).

Most patients reported that their family had a moderate or good knowledge of WD, but it was not statistically related to persistence (Table 3). In contrast, positive family support towards treatment was most commonly reported by persistent patients compared with non-persistent patients (Table 3).

## Discussion

WD is one of the few inborn metabolic diseases which can be successfully treated with pharmacological agents, but intake of medicines should continue over the whole patient’s lifespan after diagnosis to effectively reverse copper overload [1–3, 22, 23]. Unfortunately, there are many observations implying that in chronic diseases, such as diabetes or hypertension, the concordance with drug treatment is generally much lower than doctors assume [13–15, 23], but such data in WD are very limited [24]. Our current study documented that the rate of persistence, i.e., patients regularly taking pharmacological treatment without breaks, is about 74.1% in WD. These findings are consistent with treatment persistence rates in a small study of patients with WD (*n* = 39) [25] and in other chronic neurodegenerative disorders, such as Parkinson’s disease (PD) [26]. Taken together, in PD and WD, it appears that almost 25–30% of patients suffering from chronic neurological disorders are not persistent with treatment. Additionally, here we document that treatment persistence in WD has a significant positive impact on clinical treatment outcome. In patients taking medicines regularly, the persistent group, clinical improvement or disease stabilization was noted in almost 98% cases. Despite the correct anti-copper therapy, we found that 2% of patients deteriorated, probably due to other factors, such as the effects of additional drugs (hepatotoxicity, influencing dopamine neurotransmission, etc.), and this is concordant with our other studies [27]. In contrast to the situation in persistent patients, clinical worsening was noted in 52.2% of non-persistent patients. Most of the remaining patients exhibited disease stabilization (45.5%) and one patient (2.3%) showed improvement, which may be due to treatment taken between the breaks. Based on such results, we would like to emphasize that the effect of treatment persistence is a key factor related to treatment outcome in WD. Further, our data may explain, at least in part, some of the worsening of clinical symptoms or “lack of treatment efficacy” that is seen during WD treatment [7, 17, 28].

Analyzing the factors that may be related to treatment persistence, we did not find any impact of gender, type of WD treatment, phenotypic presentation, AE occurrence or duration of WD treatment. We did not confirm observations from other studies regarding treatment persistence, such as hypertension studies, which suggest that male gender is a risk factor for non-persistence [13]. We also did not detect any effect of AE occurrence on persistence. This may be explained by the mild AEs observed in our study. Skin and gastrointestinal reactions were not serious and, although they could have caused some discomfort, they did not lead to drug withdrawal. The phenotypic presentation and duration of treatment have no effect on persistence, unlike a supportive family position towards treatment. Thus, it appears that not the signs and symptoms, but family support is an important treatment persistence driver in WD. These observations are consistent with current knowledge about the significance of patient awareness to disease and persistence [12, 13]. Previously, we have shown that patients who were non-persistent with WD treatment less frequently achieved upper/post-secondary or higher education compared with persistent patients (66.0% vs. 76.3%; NS) and their primary source of outcome was significantly less often a salary (18.9% vs. 40.3%; *p* = 0.001) [29]. Finally, analyzing treatment persistence and clinical outcome, we showed that the question of which medicine to prescribe, D-penicillamine or zinc salts, is likely less important than whether the medication will be used persistently. We did not find any difference in treatment effectiveness between D-penicillamine and zinc sulphate in the patients analyzed, which is consistent with our previous observations [2, 5, 7, 17, 24, 30]. Both drugs are administered three times a day. Certainly, more advanced pharmaceutical formulations intended for once daily use may improve compliance and persistence [31, 32].

Our study has some limitations. Firstly, it is a retrospective study based mainly on subjective self-reported patient reports with secondary objective verification by the treating physician, but without separate analysis of copper metabolism. We excluded patients who were transplanted or who died from the analysis. They mostly consisted of patients severely affected since diagnosis, and reliable assessment of their persistence was not feasible. We did not include pre-symptomatic individuals since they were assessed in a previous investigation [17]. A strength of our current study is that we included patients with a WD diagnosis verified in our clinic who remained within our care. We performed only persistence analysis without the compliance analysis with accurate drugs dose calculating, like in clinical trials, because such analysis is only possible in prospective studies with drug dispensation and calculation of compliance on each visit. Also, direct analysis of compliance using a Country Prescribed Drug Register employed in some European countries [13] was not possible because no such data are available for physicians in Poland. We also did not analyze treatment adherence, meaning taking medication as prescribed in relation to the time of intake and meals, because we did not have enough objective data to verify patients’ answers. We did not assess economic status since the costs of drugs for WD are low and all Polish citizens are covered by medical insurance, with unrestricted access to necessary medical care. When analyzing factors that may impact treatment persistence, we did not analyze the effect of mental disorders. There are data that treatment persistence in patients with severe mental disorders is especially low (20–30% over a treatment period of more than 6 months) [12]; however, due to the low number of patients in our study, such an analysis of a sub-group of patients with severe neuropsychiatric impairment was not feasible. Finally, we cannot exclude the possibility that some of our patients did not record accurate responses about persistence, but physician verification of patient records aimed to reduce the likelihood of this occurring.

## Conclusions

In conclusion, our data show that treatment persistence is a key factor for a positive clinical outcome over the course of treatment for WD. It appears that the type of treatment to prescribe is less important than the fact that the medication is used persistently. Higher education and a positive, supportive family position toward WD treatment seem to be the most important factors that may impact the correct administration of de-coppering medicines by WD patients.

## Data Availability

The data sets generated and/or analyzed during this study are not publicly available because they contain protected health information. However, de-identified data sets are available from the corresponding authors on reasonable request.

## Abbreviations

AE: Adverse event
PD: Parkinson’s disease
SD: Standard deviation
WD: Wilson’s disease
